# Phytochemical Analysis, Antioxidant Activity, and Anticancer Potential of *Afzelia quanzensis* Welw—Bark Extract: A Traditional Remedy Utilized by Indigenous Communities in KwaZulu-Natal and Eastern Cape Provinces of South Africa

**DOI:** 10.3390/ijms26157623

**Published:** 2025-08-06

**Authors:** Siphamandla Qhubekani Njabuliso Lamula, Thando Bhanisa, Martha Wium, Juliano Domiraci Paccez, Luiz Fernando Zerbini, Lisa V. Buwa-Komoreng

**Affiliations:** 1 Infectious Diseases and Medicinal Plants, Botany Department, Faculty of Science and Agriculture, University of Fort Hare, Private Bag X1314, Alice 5700, South Africa; 201927865@ufh.ac.za (T.B.); lbuwa@ufh.ac.za (L.V.B.-K.); 2 International Centre for Genetic Engineering and Biotechnology (ICGEB), Weirner & Beit Building, Anzio Rd, Observatory, Cape Town 7935, South Africa; mariet.wium@icgeb.org (M.W.); juliano.paccez@icgeb.org (J.D.P.); luiz.zerbini@icgeb.org (L.F.Z.)

**Keywords:** *Afzelia quanzensis*, anticancer activity, antioxidant activity, DPPH, NO scavenging, FTIR analysis, HPLC-DAD analysis, phytochemical screening

## Abstract

Despite the significant advancements in treatment and prevention, the fight against cancer is ongoing worldwide. This study evaluated the pharmacological properties and anticancer activity of *Afzelia quanzensis* bark, traditionally used by the indigenous communities of KwaZulu Natal and Eastern Cape Provinces of South Africa to treat cancer and related illnesses. Phytochemical screening, high-performance liquid chromatography–diode array detection (HPLC-DAD), and Fourier-transform infrared spectroscopy (FTIR) analyses were carried out using established protocols. The antioxidant activity was assessed via the 2,2-diphenyl-1-picrylhydrazyl (DPPH) scavenging capacity and nitric oxide radicals. The anticancer activity was evaluated using the MTT assay (3-(4,5-dimethylthiazol-2-yl)-2,5-diphenyltetrazolium bromide). Phytochemical analysis revealed the presence of saponins, flavonoids, terpenoids, alkaloids, steroids, cardiac glycosides, and phlobatannins. The HPLC-DAD analysis detected seven distinctive peaks in the aqueous extract and three distinctive peaks in the methanolic extract. The FTIR spectra of the aqueous extract displayed characteristic peaks corresponding to O-H, C=O, C=C, and =C–H functional groups. Among the tested extracts, the methanol extract exhibited the strongest antioxidant activity, followed by the ethanolic extract, in both DPPH and nitric oxide. The methanol extract showed a higher cell proliferation inhibition against the DU-145 cancer cell line with the percentage of inhibition of 37.8%, followed by the aqueous extract with 36.3%. In contrast, limited activity was observed against PC-3, SK-UT-1, and AGS cell lines. The results demonstrated notable dose-dependent antioxidant and antiproliferative activities supporting the ethnomedicinal use of *Afzelia quanzensis* bark in cancer management. These findings warrant further investigation into its bioactive constituents and mechanisms of action.

## 1. Introduction

Cancer continues to be one of the leading causes of death globally [1]. The disease is characterized by the uncontrolled proliferation and growth of abnormal cells that form malignant tumors with the potential to metastasize [2]. According to the literature, 17.5 million new cases were reported globally in 2015, and this number is expected to increase to 23.6 million cases by 2030 [3]. Furthermore, in 2020, GLOBOCAN reported 10.3 million cancer-related deaths and 19.3 million new cases globally [4]. Despite significant advancements in treatment and prevention, cancer remains an aggressive killer worldwide [5]. Numerous cancer treatment modalities including chemotherapy, radiation, surgery and immunotherapy have significantly improved patient outcomes [6]. However, these treatments are often associated with severe side effects such as immunosuppression, bone marrow toxicity, alopecia, epithelial damage, cardiotoxicity, and neurological complications [6]. This has reinforced the ongoing need for novel anticancer agents, particularly from natural product sources [7].

The Cancer Association of South Africa (CANSA) estimates that approximately 115,000 South Africans are diagnosed with cancer annually, with prostate, colorectal, and lung cancers being the three most common cancer types among men, whereas breast and cervical cancer are the most common in women [5]. Cancer is one of the leading causes of mortality in the country, accounting for 10% of all fatalities of national deaths [8]. The South African National Cancer Strategic Framework (2017–2022) recommended an integrated approach to cancer prevention and interventions aligning with other non-communicable diseases (NCDs) due to shared risk factors [8].

South Africa possesses an abundance of diverse flora, with more than 23,000 plant species [5]. Approximately, 70% of the anticancer drugs found on the global market are derived from plants [5,9,10]. Traditional medicine remains a vital part of healthcare with about 45% of South Africans relying on it for medical care [11,12], yet there is limited scientific evidence supporting the usage of South African medicinal plants to cure cancer. Of ~250,000 higher plants found worldwide, only 5–15% have been investigated for bioactive compounds [13]. This indicates that there is still a great opportunity to explore the untapped flora for more bioactive compounds which might be useful in the future for the management of cancer and different illnesses.

Although not well documented, bark plays a significant role in traditional South African medicine. According to reports by Williams et al. [14] and Grace et al. [15], barks make around one-third of the South African medicinal plant products traded (estimated at ZAR 270 million per annum; USD 15 million) and utilised in traditional South African healthcare, which is consulted by most people [15,16]. This number might have increased overtime. *Afzelia quanzensis* Welw (family Fabaceae), also commonly known as pod mahogany or lucky bean tree, is being illegally harvested at an alarming rate in numerous African locations, including South Africa, and its population is quickly declining [17]. However, the decline in South Africa is not because of medical use, but rather land use and an increase in woodcarvings [17]. Known as “*umdlavuza*” in IsiZulu language, which means “cancer” in English, the *A. quanzensis* bark is amongst the most traded and highly valued medicinal plant parts in the KwaZulu-Natal *Muthi* market and in some parts of the Eastern Cape province. The bark of the plant is named after cancer because it is mainly used by the indigenous people of KwaZulu-Natal and Eastern Cape provinces to manage the disease.

Other than cancer treatment, the tree is also utilized for various purposes in both the commercial sector (building, making plywood, furniture, panelling and for flooring, corner poles for fencing, and railway sleepers) and traditional medicine (treatment and management of bilharzia, and eye problems) [18]. In South Africa, in addition to the aforementioned use, the tree is extensively utilized for medicinal purposes. However, this plant’s medicinal potential remains underexplored [19]. Nevertheless, the bark has previously been extracted for assessing the antibacterial activity of the green synthesized silver nanoparticles [20]. Fourier-transform infrared (FTIR) analysis revealed the presence of alkyl, carboxylates, carbonyl, and amide I, amide II, and amine functional groups, whereas the antibacterial activity showed significance growth inhibitions of *Staphylococcus aureus* and *Escherichia coli* [20].

The present study aimed to evaluate and validate the phytochemical, antioxidant, and anticancer properties of the *A. quanzensis* bark widely used to treat cancer and related illnesses.

## 2. Results

### 2.1. Phytochemical Analysis

The qualitative phytochemical screening of *A. quanzensis* bark revealed the presence of alkaloids, steroids, terpenoids, flavonoids, saponins, phlobatannin, tannins, and cardiac glycosides (Table 1).

### 2.2. FTIR Spectroscopic Analysis

**Figure 1:**
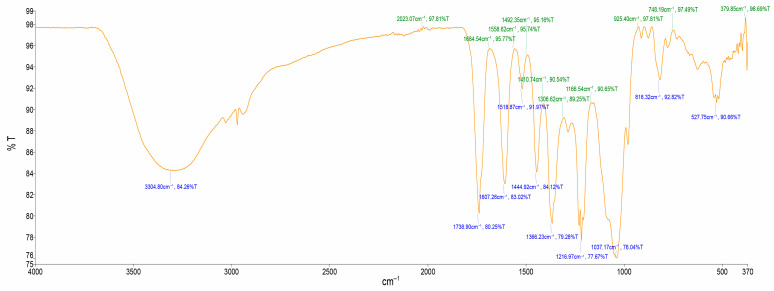
FTIR spectrum of aqueous extract from *A. quanzensis* bark; %T: Transmittance (%); cm^−1^: Wavenumber (cm^−1^).

The FTIR spectrum for the aqueous extract displayed characteristic peaks at 3304.80 cm^−1^ for O-H stretch, at 1738.90 cm^−1^ for C=O stretch, at 1444.92 cm^−1^ for C=C stretch, and at 816.32 cm^−1^ for =C–H. Figure 1 and Table 2 illustrate the quantity of absorption bands, each corresponding to distinct peaks that signify various functional groups present in a molecule.

### 2.3. HPLC-DAD of A. quanzensis Bark

**Figure 2:**
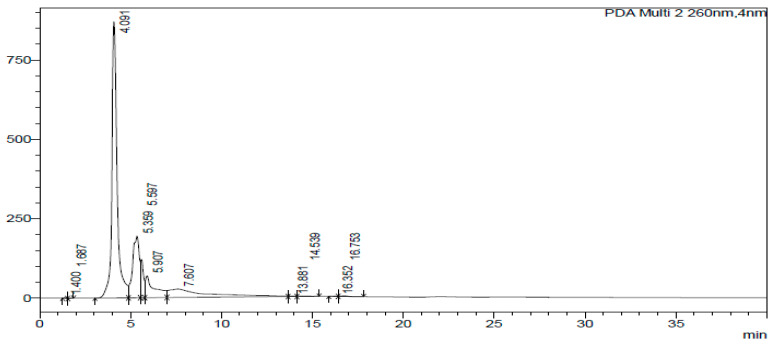
Chromatogram of water extract at 260 nm.

**Figure 3:**
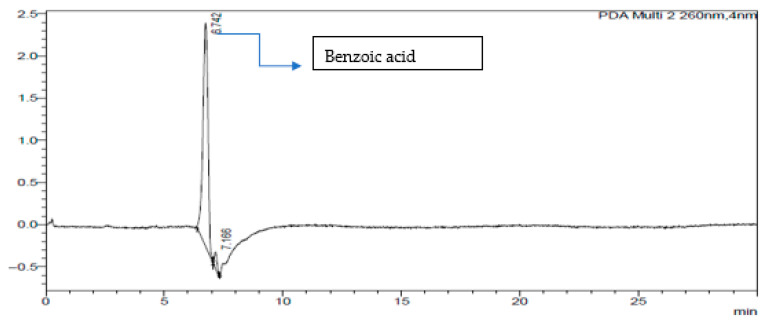
Chromatogram of water extract at 260 nm.

**Figure 4:**
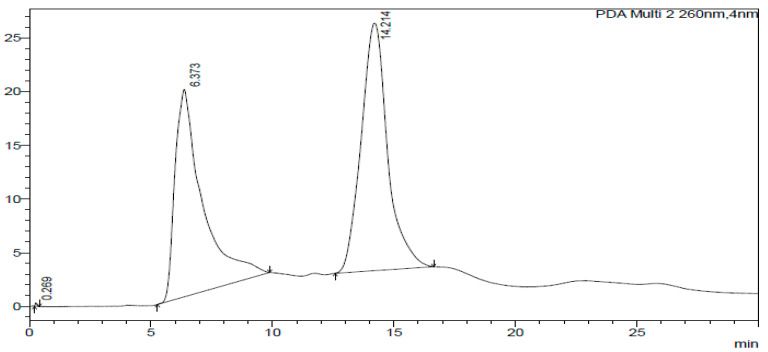
Chromatogram of ethanol extract at 260 nm.

**Figure 5:**
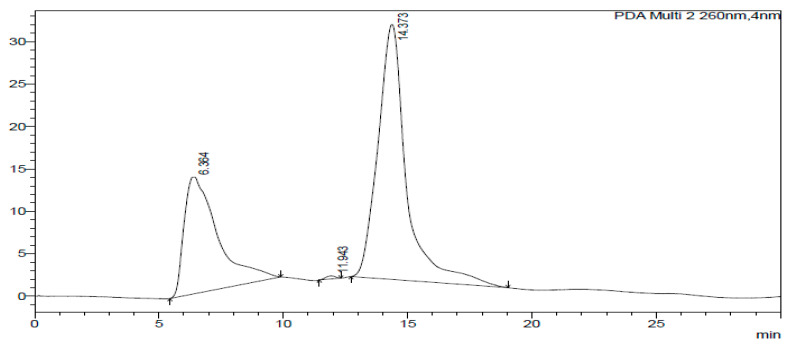
Chromatogram of methanol extract at 260 nm.

**Figure 6:**
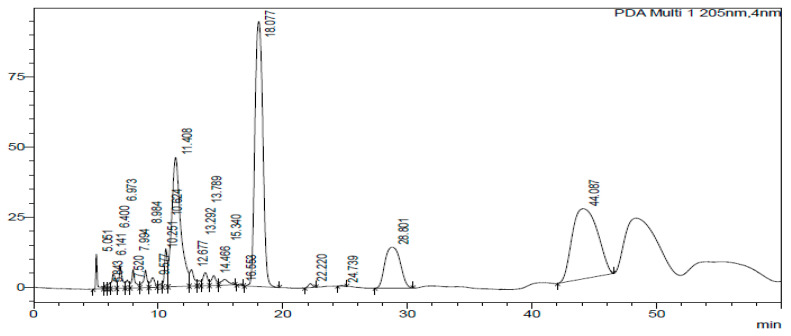
Chromatogram of hexane extract at 205 nm.

**Figure 7:**
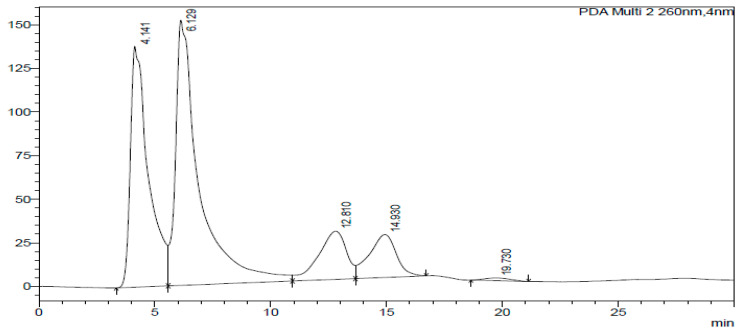
Chromatogram of ethyl acetate extract at 260 nm.

The HPLC-DAD chromatogram was generated to show the peaks that correspond to different compounds in the sample. The HPLC assay results are presented in Figure 2, Figure 3, Figure 4, Figure 5, Figure 6 and Figure 7. These graphs show peaks that might be responsible for the biological activities. Benzoic acid was used as a standard compound. Figure 2 shows the unique pattern of peaks and their corresponding UV/Vis spectra under 30 min. Using the ethanol extract, the peak at 14.214 min in Figure 4 indicates that there was successful plant extraction, whereas, using the methanol extract, Figure 5 shows a successful extraction at 14.373 min Rt and identification at 6 min Rt. Several peaks observed in Figure 6 indicate that plant extraction was successful. However, the constituents in the extract were not adequately separated by the HPLC conditions. The peaks at 4.141, 12.810, 14.930, 19.730 min Rt in Figure 7 indicate that plant extraction was successful using ethyl acetate extract.

### 2.4. Antioxidant Assay

**Figure 8:**
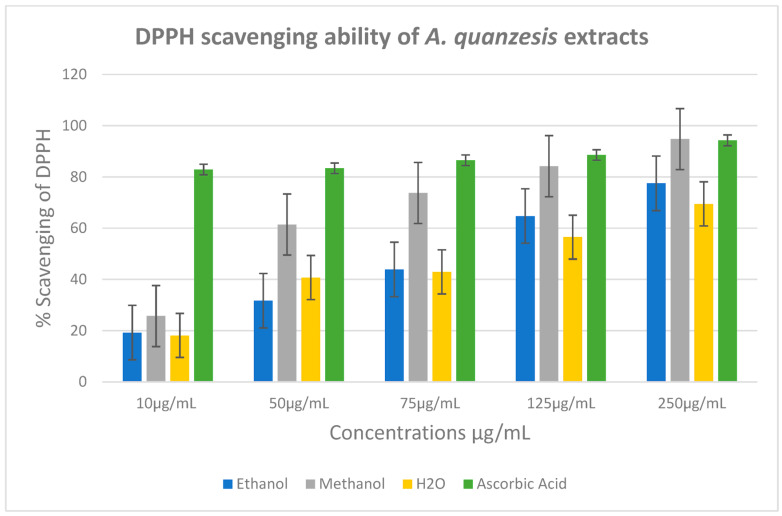
% DPPH radicals’ scavenging activity of ethanol, methanol and aqueous extracts.

The percentage of scavenging of DPPH of the extracts was determined for aqueous, ethanol, and methanol extracts as 69.6%, 77.5%, and 94.9%, respectively (Figure 8). As shown in Figure 8, the methanol extract exhibited the highest antioxidant properties when compared to ascorbic acid, a standard antioxidant at 250 µg/mL. Comparing this with the percentage of scavenging of DPPH of ascorbic acid of 94 µg/mL, only methanol had a close value of ascorbic acid. The lC50 for the aqueous, ethanol, and methanol extracts were determined to be 3 ± 17, 3 ± 21 and 2 ± 24 μg/mL, respectively, whereas ascorbic acid had the lC50 value of 2 ± 4 μg/mL (Table 3).

**Figure 9:**
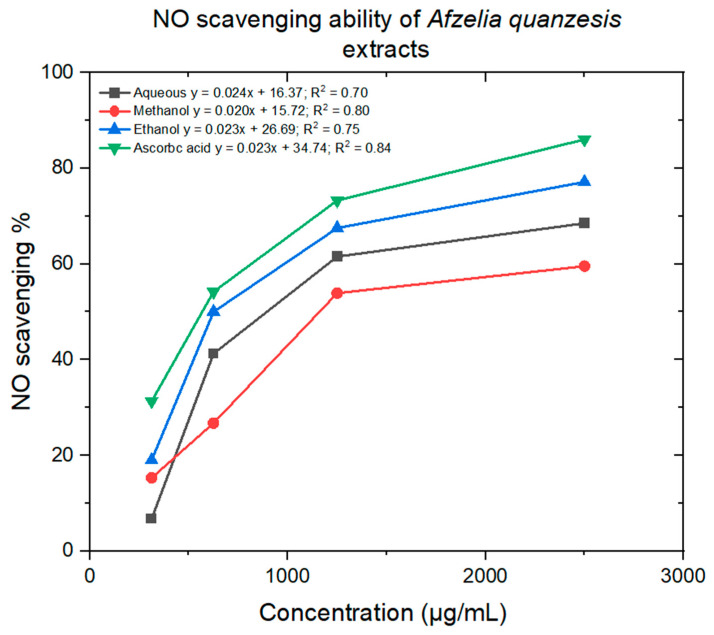
NO scavenging activity of ethanol, methanol, and aqueous extracts.

Figure 9 illustrates a minimal decrease in the NO radical due to the scavenging ability of the aqueous, ethanol, and methanol extracts, as well as ascorbic acid. The methanol, aqueous, and ethanol extracts showed minimum lC50 values of 1714 ± 16, 1401 ± 10 and 10,134 ± 14 μg/mL, respectively, whereas ascorbic acid had an lC50 value of 664 ± 10 μg/mL (Table 3).

### 2.5. Anticancer Activity

**Figure 10:**
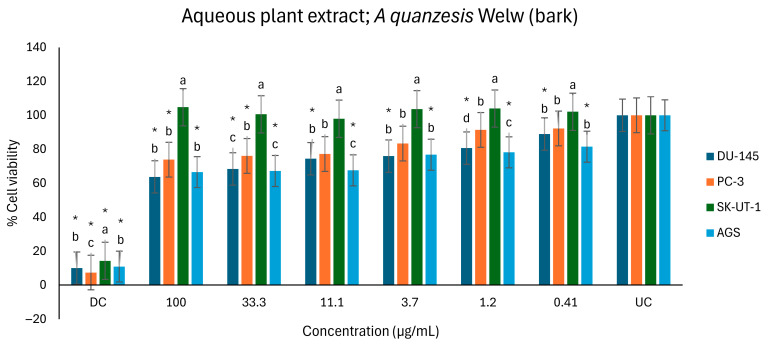
Antiproliferative effects of *A. quanzensis* bark aqueous extract against selected cancer cell lines (UC and DC mean untreated control and drug control, respectively) as determined by the MTT assay. Means are an average of six concentrations for each extract ± SD. Error bars with different letters are significantly different (*p* < 0.05). Asterisk represents a significant difference from the untreated control.

**Figure 11:**
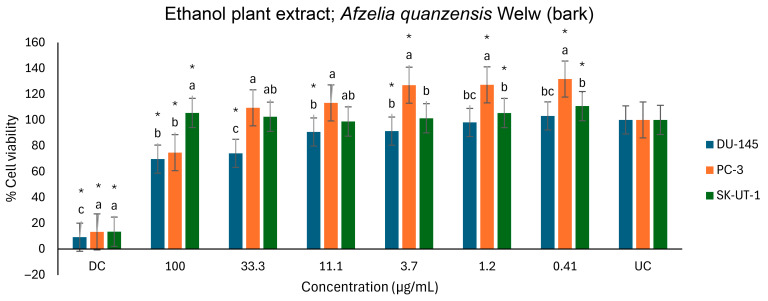
Antiproliferative effects of *A. quanzensis* bark ethanol extract against selected cancer cell lines (UC and DC mean untreated control and drug control, respectively) as determined by the MTT assay. Means are an average of six concentrations for each extract ± SD. Error bars with different letters are significantly different (*p* < 0.05). Asterisk represents a significant difference from the untreated control.

**Figure 12:**
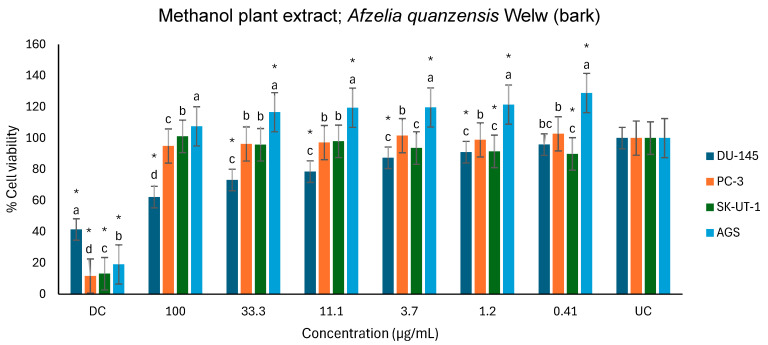
Antiproliferative effects of *A. quanzensis* bark methanol extract against selected cancer cell lines (UC and DC mean untreated control and drug control, respectively) as determined by the MTT assay. Means are an average of six concentrations for each extract ± SD. Error bars with different letters are significantly different (*p* < 0.05). Asterisk represents a significant difference from the untreated control.

**Figure 13:**
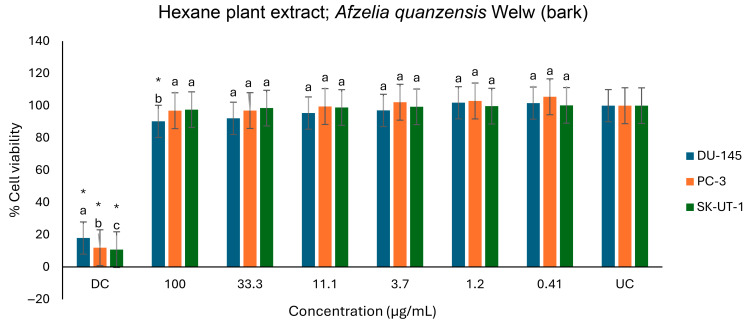
Antiproliferative effects of *A. quanzensis* bark hexane extract against selected cancer cell lines (UC and DC mean untreated control and drug control, respectively) as determined by the MTT assay. Means are an average of six concentrations for each extract ± SD. Error bars with different letters are significantly different (*p* < 0.05). Asterisk represents a significant difference from the untreated control.

Table 4 shows the results of the percentage of inhibition of plant extracts on cancer cells as determined by the MTT assay. The effects of the inhibition of aqueous, ethanol, methanol, and hexane extracts in *A. quanzensis* bark on DU-145, PC-3, SK-UT-1, and AGS cell lines are shown in Figure 10, Figure 11, Figure 12 and Figure 13. The untreated cells were used as a negative control, while the docetaxel (a taxane-based chemotherapy drug) was used as a drug control.

Although the aqueous extract exhibited moderate activity, it was less potent than the positive control, docetaxel. However, compared to the untreated control, it demonstrated moderate dose-dependent activities against the AGS and DU-145 cell lines across all concentrations tested, indicating dose dependency (Figure 10). Compared to the untreated control, the extract showed moderate activity against the viability of the PC-3 cell line at concentrations ranging from 11.1 to 100 µg/mL, signifying dose-dependent anticancer potential, especially at higher concentrations. Conversely, the aqueous extract showed minimal to no anticancer activity against the SK-UT-1 cell line across all concentrations tested.

The percentage of inhibition of DU-145 and PC-3 was 36.3% and 26.1% at 100 µg/mL, respectively (Table 4).

Figure 11 also depicts the anticancer activities of the ethanol extract on three cell lines. However, compared to the untreated control, several significant activities were observed. The ethanol extract showed a significant effect on the viability of the DU-145 cells, in a dose-dependent manner, from 33.3 to 100 µg/mL. At the highest concentration (100 µg/mL) tested, the ethanol extract moderately reduced the PC-3 cells’ viability. However, the extract appears to have proliferative effects at the lower concentrations (0.41 to 3.7 µg/mL) as the cells’ viability increased beyond that of the UC. A similar moderate proliferative effect was found on the SK-UT-1 cells, as well, at 0.41, 1.2, and 100 µg/mL. The ethanol extracts inhibited DU-145 and PC-3 cell lines by 30.4% and 25.3% at 100 µg/mL, respectively.

Figure 12 shows the anticancer activities of the methanol extract on the DU-145, PC-3, SK-UT-1 and AGS cell lines. l. Compared to the untreated control, the methanol extract exhibited a significant effect on the viability of the DU-145 cell line, in a dose-dependent manner, from 1.2 to 100 µg/mL. In contrast, the extract exhibited a minimal effect on the viability of the PC-3 and SK-UT-1 cell lines. Interestingly, the extract displayed a significant proliferative effect on the AGS cell line from 0.41 to 33.3 µg/mL and strangely had no significant effect at the highest concentration (100 µg/mL) tested. Methanol extract also demonstrated a dose-dependent activity against DU-145 with a percentage of inhibition of 37.8% at 100 µg/mL (Table 4 and Figure 12).

The hexane exhibited limited anticancer activity on the DU-145, PC-3, and SK-UT-1 cell lines, and showed no significant effect on the viability of three cell lines compared to the drug control (Figure 13). Compared to untreated control, the extract only exhibited a moderate effect on the viability of the DU-145 cell line at the highest concentration of 100 µg/mL. Consequently, the hexane extract displayed the lowest anticancer activity among all the extracts tested.

## 3. Discussion

### 3.1. Phytochemical Analysis

The phytochemical analysis of *A. quanzensis* bark revealed the presence of alkaloids, steroids, terpenoids, flavonoids, saponins, phlobatannin, tannins, and cardiac glycosides. According to a study by Burlacu et al. [21], the chemical composition of the majority of the bark extracts is made up of bioactive compounds that are polyphenols, alkaloids, terpenoids, carbohydrates, proteins, saponins, and vitamins. The presence of polyphenols (flavonoids and tannins) has been demonstrated to exhibit anticancer effects against several epithelial cancers [22]. According to the review by Raina et al. [23], terpenoids found in *Boswellia serrata* Roxb demonstrated the ability to strongly inhibit tumour angiogenesis induced through vascular endothelial growth factor (VEGF) signalling. It further inhibits multiple steps of VEGF-induced cell proliferation, migration, invasion, and tube formation; Active alkaloids and its derivatives present in *Catharanthus roseus* (L.) are well known for their significant curative effects against human neoplasms; Glycosides and its derivative present in *Centella asiatica* L. has shown to decrease the viability of HepG2 cells in the case of liver cancer and Saponins, steroids and its derivatives present in *Panax ginseng* has demonstrated antitumor potential and a potential to induce cell death. The presence of tannins in medicinal plants has been suggested to be useful as an anti-diarrheic and antihemorrhagic agent [24].

### 3.2. FTIR Spectroscopic Analysis

The FTIR analysis (Table 2) revealed the presence of polyphenols and flavonoids due to O-H stretching, terpenes due to C-H group [25]. The functional groups present in *A. quanzensis* bark are phenols, carboxyl group, ketones, aromatic compound, amine, phosphate, alkene and alkyl halides. These results are similar to the findings of Moyo et al. [20]. All these compounds belong to secondary plant metabolites and could be responsible for various medicinal properties of the plant [26,27].

### 3.3. HPLC-DAD Analysis of A. quanzensis Bark Extracts

The HPLC analysis of different extracts contained major peaks along with many small peaks indicating the presence of active major compounds [28]. The methanol extract showed clear distinct peaks, followed by the ethanol extract. The water and hexane extracts contained several peaks which showed successful extractions. However, these peaks show that the constituents in the extract were not adequately separated by the HPLC conditions. This might be due to compounds within the extracts working in synergy. The HPLC results are consistent with both the phytochemical and FTIR results. The small peaks may be attributed to the compounds present in small quantities as well as disintegrated major compounds [28]. The peaks related to low retention times are mainly low polar plant compounds. Nuclear magnetic resonance (NMR) is needed to identify the compounds. NMR helps in the isolation and characterization of bioactive compounds from plants and will assist in identifying and quantifying specific compounds within the extract, even in complex mixtures. It can also be used to verify the identity, purity, and composition of plant extracts, ensuring the quality of botanical products.

### 3.4. Antioxidant Assay

The antioxidant activity of the medicinal plant was studied by monitoring the ability of the plant extracts to scavenge free radicals generated in vitro. The aqueous, ethanol, and methanol extracts of *A. quanzensis* bark were examined for their ability to scavenge DPPH radical. All the extracts demonstrated dose-dependent DPPH scavenging activities. Methanol extract exhibited the highest antioxidant properties when compared to ascorbic acid, a standard antioxidant. According to Kandawa-Schulz et al. [9], methanol extracts tend to show higher antioxidant activity compared to other extracts. There are studies which have described the antifungal activity [29], antibacterial and antioxidant activity of *A. quanzensis* [21].

Nitric oxide prevents cell death caused by H_2_O_2_, alkylhydroperoxides, and xanthine oxidase and appears to limit oxidative harm to mammalian cells primarily through the inhibition of metal/peroxide oxidative chemistry and lipid peroxidation. In addition to these chemical and biochemical properties, NO can influence cellular and physiological processes to prevent oxidative injury, including leukocyte adhesion [30]. In this study, the scavenging activity of NO by methanol, aqueous, and ethanol extracts increased in dose-dependent manner. Methanol and aqueous extracts showed a minimal NO scavenging activity, whereas the ethanol extract demonstrated a high NO scavenging activity when compared to ascorbic acid (Figure 9).

### 3.5. Anticancer Activity

Medicinal plants continue to play a critical role in modern medicine, especially in developing countries. It is estimated that over 75% of plant-derived compounds presently in use worldwide are a result of studies that aim to verify the authenticity of data from folk and ethnomedical uses based on traditional practices [31]. The majority of important drugs found in the market today have been derived from plants [32]. Bark products constitute nearly one third of plant material used in South African traditional medicine [15]. *A. quanzensis* bark is among the most used medicinal plant species to treat different ailments. In South Africa it is commonly used to treat cancer.

PC-3 and DU-145, both prostate cancer cell lines that are androgen receptor-independent. They differ in terms of their tumorigenic and metastatic potential. For example, when injected into an immuno-compromised mouse, PC-3 cell line forms highly metastatic grade IV adenocarcinoma. In contrast, the DU-145 cell line develops prostate cancer with moderate metastatic potential [33]. The human prostate cancer is the second cause of cancer death in men [34]. Uterine leiomyosarcoma (SK-UT-1), is a rare malignant smooth muscle tumour originating in the uterine wall that generally responds poorly to chemotherapy and radiation. It is a malignant tumour accounting for about 40% of all uterine sarcomas [35]. Gastric cancer (AGS) is a type of gastrointestinal tract cancer that is the greatest cause of cancer-related mortality in the world. Around 90% of stomach cancers are adenocarcinomas [36].

*Afzelia quanzensis* bark extracts revealed the strong presence of flavonoids, terpenoids, and alkaloids which have been documented to possess anticancer activities [1,37]. All the extracts demonstrated a dose-dependent activity against the DU-145 and PC-3 cancer cell lines. The aqueous extract showed significant effect against the DU-145 and moderate activity against PC-3 cell line. Water is usually the universal solvent used by traditional or indigenous people to prepare medication. The aqueous extract had the highest anticancer activity compared to the other extracts, while the methanol extract demonstrated the highest efficacy only against the DU-145 cell line. Ethanol, like the aqueous extract, had a strong dose-dependent effect on the viability of DU-145 cells and moderately affected the viability of PC-3 cells. Ethanol is often used as a solvent to dissolve plant extracts because it can dissolve a wide range of compounds, including both polar and nonpolar substances [37]. Ethanol is a secure and efficient method for extracting oils and chemicals from plants, frequently employed in extraction techniques such as maceration, percolation, and Soxhlet extraction. However, with plant extracts, it is difficult to conclude which compounds are responsible for the anticancer activity or whether the activity observed is caused by a single compound or a synergy of compounds working together. The methanol and hexane extracts had a lower response against the majority of cell lines, except for the methanol extract against the DU-145 cell line, which was highly effective. Methanol is a widely used solvent in plant extract research because it can dissolve both hydrophilic and lipophilic compounds, making it suitable for extracting a wide variety of phytochemicals [38]. While methanol is known for its toxicity, it is frequently employed in plant extraction to produce high levels of beneficial chemicals, including antioxidants [37]. However, traditional healers have less access to methanol, and it has been reported to cause a wide range of neurological manifestations, most commonly confusion, coma and vision loss and less frequent tremors as a manifestation of putaminal hemorrhage/necrosis [39]. Following extraction, the methanol can be simply evaporated or removed, leaving behind the required plant extract. On the other hand, hexane is often used to dissolve nonpolar plant extracts, especially in the initial stages of a multi-solvent extraction process, like a “defatting” step. It is commonly used to remove lipids and other nonpolar compounds before using solvents with higher polarity and it extracts the lowest mass of plant material, and this might be the reason most studies record the least activity [38]. This is in contradiction with the finding by Baskar et al. [31], which showed that methanol and hexane had the highest antiproliferation against cancer cell lines compared to other extracts. Saleh et al. [40] found that secondary metabolites in the hexane extract demonstrated the highest cytotoxicity, and thus anticancer activity, against HCT-116 cells, with an IC50 of 17.15 ± 0.78 mg/mL. In general, polar solvents such as water, methanol, and ethanol are used in extraction of polar compounds, whereas nonpolar solvents such as hexane and dichloromethane are used in extraction of nonpolar compounds [38]. All the extracts demonstrated the least response against SK-UT-1. Similarly, the aqueous and methanol extract exhibited the least response against AGS. Cancer cell lines also tend to react differently to different plant extracts [41]. Nevertheless, further investigation is required to comprehensively ascertain the therapeutic potential of *A. quanzensis* in cancer treatment.

## 4. Materials and Methods

### 4.1. Collection of Plant Material

The *A. quanzensis* bark was purchased from the local *Muthi* market in Durban, KwaZulu-Natal province of South Africa. Additional bark material was obtained from personnel working in nature conservation. Prior identification (succession number: SA Tree No. 207) was carried out by a taxonomist, at the Faculty of Science and Agriculture, University of Fort Hare, South Africa.

### 4.2. Preparation of Extracts

The extraction was performed using five solvents of increasing polarity (hexane, acetone, ethanol, methanol, and aqueous). Thirty grams (30 g) of the dried, powdered material was extracted in 300 mL of ethanol, methanol, hexane and distilled water, respectively, by shaking on Labcon platform shaker (Laboratory Consumables, PTY, Durban, South Africa) for 24 h at room temperature. Extracts were filtered through Whatman No. 1 filter paper. Ethanol, methanol, and hexane extracts were concentrated under reduced pressure at 45 °C using a rotary evaporator (Cole Parmer SB 1100, Shanghai, China) whereas the filtrate from the water extract was evaporated to dryness using a freeze-dryer (Genevac LTD, BTP-3ES00X, Ipswich, England). All the crude extracts were stored at −20 °C until use.

For anticancer assays, stock solutions were prepared by dissolving 0.04 g crude ethanol, methanol and hexane extracts in 2 mL dimethyl sulfoxide (DMSO), while the crude water extract was dissolved in 2 mL distilled water. All extracts were vortexed and filtered through 0.45 μm and 0.22 μm sterile filters under sterile conditions. The filtered aliquot extracts were wrapped with foil and stored at −20 °C until use.

### 4.3. Qualitative Phytochemical Screening

The qualitative phytochemical analysis of *A. quanzensis* bark was determined by adopting the standard methods described by Harborne [42], Trease and Evans [43], Sofowora [44] and Edeoga et al. [45]. The bark was tested for the presence of alkaloids [42], flavonoids [44], terpenoids [42], saponins [42], anthraquinones [42], cardiac glycosides [43], and tannins [44]. The presence of phytochemicals was determined by visually observing colour change or the production of a precipitate upon the addition of the prescribed reagent(s). The experiment was performed in triplicates.

### 4.4. Fourier-Transform Infrared Spectroscopy Analysis

The Fourier-transform infrared spectroscopy (FTIR) analysis was performed to identify the functional groups present in the plant’s extracts and to indicate the presence of these functionalities on the phytochemical compounds. Both dried powder and water extract of the plant material were analysed. FTIR was performed on the translucent sample disc. Then, 10 mg of each sample (the crude aqueous extract and powdered plant material) were combined with 100 mg of potassium bromide (KBr) pellet and each loaded into the FTIR spectroscope (Perkin Elmer Spectrum 100 FTIR spectrometer), PerkinElmer, 710 Bridgeport Ave Shelton, CT, USA. The FTIR spectroscope’s scan range was set from 400 to 4000 cm^−1^ with a resolution of 4 cm^−1^ for accurate analysis. The analysis was performed in triplicates.

### 4.5. HPLC-DAD Analysis of A. quanzensis Bark Extracts

A stock solution of water, ethanol, methanol, hexane, and ethyl acetate extracts was prepared. High-performance liquid chromatography–diode array detection (HPLC-DAD) analysis was carried out using an Agilent HPLC 1200 infinity series system, equipped with a photodiode array detector (Agilent Technologies, Waldbronn Germany). The chromatograms were recorded at 205 and 260 nm. An Agilent Zorbax Eclipse Plus C18 column (3.5 µm × 150 mm × 4.6 nm) (Agilent, Newport, CA, USA) was operated at an oven temperature of 25 °C. The mobile phase was a mixture of 30% water (mobile phase A) and 70% methanol (mobile phase C). A flow rate of 1 mL/min was used throughout the analysis. The eluate was injected into HPLC-DAD system for quantitative and qualitative analysis [46]. An OHAUS starter 2100 pH meter (Pine Brook, NJ, USA) was used for pH adjustments of the reagents and to measure the pH of the samples. The results were obtained in chromatograms showing the peaks of identified compounds. The analysis was performed in triplicate.

### 4.6. Antioxidant Assay

#### 4.6.1. DPPH Radicals Scavenging Assay

The antioxidant properties of different solvent extracts of the plant were determined by 2,2-diphenyl-1-picrylhydrazyl (DPPH) scavenging activities. A volume of 50 µL of 0.3 mM of DPPH stock solution in methanol was gently mixed with five different concentrations (250 µg/mL, 125 µg/mL, 50 µg/mL, 10 µg/mL, 5 µg/mL) of each of plant extracts and control (ascorbic acid) was also prepared in methanol. A blank sample (no concentration) and control samples were also prepared. The solution was incubated for 30 min at room temperature before the absorbance was read with a spectrophotometer at 517 nm. Antioxidant property was determined with Equation 1 below as described by Madikizela and McGaw [47]. The experiment was performed in triplicates.

% DPPH scavenging activity = (Absorbance of sample − Absorbance of blank)/(Absorbance of control − Absorbance of Blank) × 100(1)

#### 4.6.2. Nitric Oxide (NO) Scavenging Activity

The nitric oxide scavenging activity of the plant extracts was determined by the method outlined by Wintola and Afolayan [48]. Next, 2 mL of 10 mM sodium nitroprusside prepared in phosphate-buffered saline (pH 7.4) was mixed with 0.5 mL of each extract together with standard solutions BHT and gallic acid at different concentrations (50, 100, 200, 300, 400, 500 μg/mL). After the 2.5 h incubation of the samples at 25 °C, 0.1 mL of the incubated sample was combined with 0.1 mL of the Griess reagent [1.0 mL sulfanilic acid reagent (0.33%) prepared in 20% glacial acetic acid] and left at room temperature for 5 min. A 1 mL of naphthylenediamine dichloride (0.1% *w*/*v*) was added to the mixture and further incubated for 30 min at room temperature. The absorbance was read at 540 nm. The analysis was performed in triplicates. The amount of nitric oxide radicals inhibited by plant extracts was calculated using the following equation:

NO radical scavenging activity(%) = (Absorbance of sample − Absorbance of blank)/(Absorbance of control − Absorbance of Blank) × 100(2)

where Abscontrol is the absorbance of NO radicals + methanol and Abssample is the 475 absorbance of NO radical + extract or standard.

### 4.7. Anticancer Activity

Human prostate carcinoma (DU-145 and PC-3), uterine leiomyosarcoma (SK-UT-1), and gastric adenocarcinoma (AGS) cancer cell lines obtained from American Type Culture Collection (ATCC). Cell lines were maintained in Dulbecco’s Modified Eagles Medium (DMEM) containing 10% fetal bovine serum (FBS), 1 mM L-glutamine, 100 units/mL penicillin, and 100 μg/mL streptomycin and incubated at 37 °C in a humidified 5% CO_2_ incubator (Thermos Fisher Scientific, Waltham, MA, USA). Cell lines were selected based on their availability.

The plate was divided into three: background control (no cells, no treatment), cells, with no treatment (as in the 100% growth), cells in the same percentage of DMSO as the highest concentration of plant extract and cells treated with *A. quanzensis* bark extracts at different concentrations. The treatment was performed in triplicates.

The anticancer activity of the plant extracts was tested in vitro on DU-145, PC-3, SK-UT-1, and AGS cell lines using a modified MTT (3-(4,5-dimethylthiazol-2-yl)-2, 5-diphenyltetrazolium bromide) tetrazolium reduction assay as described by [49]. The ethanol and hexane extracts were tested on 3 (DU-145, PC-3, and SK-UT-1) cell lines, whereas the aqueous and methanol extracts were tested on 4 (DU-145, PC-3, SK-UT-1, and AGS) cancer cell lines. Briefly, 6 × 10^3^ cells/well in 100 μL complete media were seeded into a 96-well cell culture plate. Next, 100 µg/mL of the diluted plant extract was added to the 96-well culture plate and serial dilution performed, with untreated cell media solution as a control. The concentration ranged from 100 μg/mL to 0.41 μg/mL. The 96-well cell culture plates were then incubated at 37 °C in humidified 5% CO_2_ for 72 h. Following incubation, 10 μL MTT (2.5 mg/mL) was added to each well and incubated for another 4 h and after which the experiment was stopped by adding a sodium dodecyl sulfate 10% in 0.1 N HCl solution was added to solubilize the formed formazan and incubated overnight. Optical density in the wells was read in a microplate reader (Thermo Multiskan Go, Agilent, Santa Clara, CA, USA) at a wavelength of 595 nm [49]. The absorbance values obtained from the control (drug control and untreated cell solution) wells were averaged and this value was considered as 100% cell viability. Cell viability was calculated as follows:

Percentage cell viability = (Absorbance of sample)/(Absorbance of control) × 100%(3)

### 4.8. Statistical Analysis

Microsoft Excel 2013 Windows and OriginLab 8.6 software were used for all statistical analyses and plotting of the graphs. Data is presented as mean ± SD. The *p* < 0.05 and *p* < 0.01 were considered as statistically significant using Student’s t-test.

## 5. Conclusions

The pharmacological analysis of the *A. quanzensis* bark revealed the presence of a majority of phytochemicals and bioactive compounds that have been documented to possess therapeutic properties, including antiproliferation activities. Plant extracts exhibited dose-dependent in vitro antioxidant and antiproliferative activities. Functional groups found in the plant further confirm its therapeutic potential. However, pure compounds must be isolated, identified, and tested individually to see whether the observed activities will increase or decrease. The current study’s findings, as well as the bioactivities observed, validate traditional healers’ claims that the bark contains medicinal or therapeutic properties.

## Figures and Tables

**Table 1:** Qualitative phytochemical screening of *A. quanzensis* bark.

S/No.	Compounds	Colour Change	D(+)/ND(−)
1	Alkaloids	Yellowish–creamish	++
2	Steroids	Violet to blue	+
3	Terpenoid	Reddish brown	+
4	Flavonoids	Orange	++
5	Saponins	Frothing	+
6	Phlobatannins	Dirty green precipitate	++
7	Tannins	Red precipitate	++
8	Cardiac glycosides	Brown ring	+

(+): detected; (−): not detected; (++): strongly detected.

**Table 2:** FTIR interpretation of functional group of aqueous extract of *A. quanzensis* bark.

Spec. No	Wave Number cm^−1^ (Test Samples)	Wave Number cm^−1^ (Hemmalakshmi et al., 2017)	Functional Group
1	3304.80	3570–3200	O–H strech
2	1738.90	1820–1670	C=O stretch
3	1607.26	1650–1600	C=O stretch
4	1518.87	1600–1400	C=C stretch
5	1444.92	1600–1400	C=C stretch
6	1366.23	1410–1310	O–H stretch, alcoholic group
7	1216.97	1360–1210	C–N stretch
8	1037.17	1100–1000	PO_3_ stretch
9	816.32	1000–675	=C–H
10	527.75	730–500	C–Cl

**Table 3:** DPPH and NO scavenging activity of *A. quanzensis* bark extracts.

Samples	DPPH (lC50 μg/mL)	NO (lC50 μg/mL)
Aqueous	4 ± 17	1401 ± 10
Ethanol	3 ± 21	1014 ± 14
Methanol	2 ± 24	1714 ± 16
Ascorbic acid	2 ± 4	664 ± 10

**Table 4:** Shows the inhibitory percentages of plant extracts on cancer cells as assessed by the MTT test.

Cell Lines				
	DU-145	PC-3	SK-UT-1	AGS
**Plant Extracts**	**% Inhibition at 100 µg/mL**			
Aqueous	36.3	26.1	-	33.4
Ethanol	30.4	25.3	-	Not tested
Methanol	37.8	5.1		-
Hexane	-	-	-	Not tested
(−); No activity				

## Data Availability

The original contributions presented in this study are included in the article. Further inquiries can be directed to the corresponding author(s).

## References

[1] Masumbu F.F.F., Mwamatope B., Tembo D., Mwakikunga A., Kamanula J. (2023). Ethnobotanical Survey of Medicinal Plants Claimed by Traditional Herbal Practitioners to Manage Cancers in Malawi. J. Herb. Med..

[2] Alabi M.A., Muthusamy A., Kabekkodu S.P., Adebawo O.O., Satyamoorthy K. (2020). Anticancer Properties of Recipes Derived from Nigeria and African Medicinal Plants on Breast Cancer Cells in Vitro. Sci. Afr..

[3] Ismail M., Khan S., Khan F., Noor S., Sajid H., Yar S., Rasheed I. (2020). Prevalence and Significance of Potential Drug-Drug Interactions among Cancer Patients Receiving Chemotherapy. BMC Cancer.

[4] Chhikara B.S., Parang K. (2023). Chemical Biology LETTERS Global Cancer Statistics 2022. The Trends Projection Analysis.

[5] Twilley D., Rademan S., Lall N. (2020). A Review on Traditionally Used South African Medicinal Plants, Their Secondary Metabolites and Their Potential Development into Anticancer Agents. J. Ethnopharmacol..

[6] Hamedi A., Bayat M., Asemani Y., Amirghofran Z. (2022). A Review of Potential Anti-Cancer Properties of Some Selected Medicinal Plants Grown in Iran. J. Herb. Med..

[7] Fouche G., Cragg G.M., Pillay P., Kolesnikova N., Maharaj V.J., Senabe J. (2008). In Vitro Anticancer Screening of South African Plants. J. Ethnopharmacol..

[8] Finestone E., Wishnia J. (2022). Estimating the burden of cancer in South Africa. S. Afr. J. Oncol..

[9] Kandawa-Schulz M., El-Sayed Lofty H., Lyantagaye S. (2018). Anticancer, Antioxidant and Antimicrobial Screening of Extracts from Selected Medicinal Plants from Oshikoto, Namibia. Asian J. Trop. Biotechnol..

[10] Mlilo S., Sibanda S. (2022). An Ethnobotanical Survey of the Medicinal Plants Used in the Treatment of Cancer in Some Parts of Matebeleland, Zimbabwe. S. Afr. J. Bot..

[11] McFarlane C. (2015). South Africa: The Rise of Traditional Medicine. Insight Afr..

[12] Hughes G.D., Aboyade O.M., Okonji C.O., Clark B., Mabweazara S.Z. (2021). Comparison of the Prevalence of Non-Communicable Diseases and Traditional Herbal Medicine Use in Urban and Rural Communities in South Africa. Adv. Integr. Med..

[13] Juárez P. (2014). Plant-Derived Anticancer Agents: A Promising Treatment for Bone Metastasis. BoneKEy Rep..

[14] Williams V.L., Witkowski E.T.F., Balkwill K. (2007). Relationship between Bark Thickness and Diameter at Breast Height for Six Tree Species Used Medicinally in South Africa. S. Afr. J. Bot..

[15] Grace O.M., Prendergast H., Van Staden J., Jäger A.K. (2002). The status of bark in South African traditional health care. S. Afr. J. Bot..

[16] Cunningham T. (1989). Herbal medicine trade: A hidden economy. Indic. S. Afr..

[17] Gerhardt K., Todd C. (2009). Natural Regeneration and Population Dynamics of the Tree *Afzelia quanzensis* in Woodlands in Southern Africa. Afr. J. Ecol..

[18] PlantZAfrica. https://pza.sanbi.org/afzelia-quanzensis.

[19] van Wyk B.-E. (1997). Medicinal Plants of South Africa.

[20] Moyo M., Gomba M., Nharingo T. (2015). *Afzelia quanzensis* Bark Extract for Green Synthesis of Silver Nanoparticles and Study of Their Antibacterial Activity. Int. J. Ind. Chem..

[21] Burlacu E., Tanase C., Coman N.A., Berta L. (2019). A Review of Bark-Extract-Mediated Green Synthesis of Metallic Nanoparticles and Their Applications. Molecules.

[22] Ahmed S.I., Hayat M.Q., Tahir M., Mansoor Q., Ismail M., Keck K., Bates R.B. (2016). Pharmacologically Active Flavonoids from the Anticancer, Antioxidant and Antimicrobial Extracts of *Cassia angustifolia* Vahl. BMC Complement. Altern. Med..

[23] Raina H., Soni G., Jauhari N., Sharma N., Bharadvaja N. (2014). Phytochemical Importance of Medicinal Plants as Potential Sources of Anticancer Agents. Turk. J. Bot..

[24] Xaba V.M., Adeniran A.L., Lamula S.Q.N., Buwa-Komoreng L.V. (2024). In Vitro Bioactivities of Plants Used against Skin Diseases in the Eastern Free State, South Africa. Int. J. Plant Biol..

[25] Kalaichelvi K., Dhivya S.M. (2017). Screening of Phytoconstituents, UV-VIS Spectrum and FTIR Analysis of *Micrococca mercurialis* (L.) Benth. Int. J. Herb. Med..

[26] Durak T., Depciuch J. (2020). Effect of Plant Sample Preparation and Measuring Methods on ATR-FTIR Spectra Results. Environ. Exp. Bot..

[27] Bhawsar J., Jain P.K., Soni A., Jain P. (2016). Phytochemical Analysis of *Mentha spicata* Plant Extract Using UV-VIS, FTIR and GC/MS Technique. J. Chem. Pharm. Res..

[28] Chaudhry A.W., Memon A.A., Mangi J.U., Gorar M., Zaman N., Mahar Z.A., Soomro S.A., Qureshi A.U., Sidhu A.R. (2024). An Efficient Determination of Phenolic Compounds by HPLC-Dad and Their Bioactivity Assay from Aerial Parts of *Eucalyptus tereticornis*. Pak. J. Bot..

[29] Steenkamp V., Fernandes A.C., Van Rensburg C.E.J. (2007). Screening of Venda Medicinal Plants for Antifungal Activity against *Candida albicans*. S. Afr. J. Bot..

[30] Mittal M., Siddiqui M.R., Tran K., Reddy S.P., Malik A.B. (2014). Reactive Oxygen Species in Inflammation and Tissue Injury. Antioxid. Redox Signal..

[31] Baskar A.A., Al Numair K.S., Alsaif M.A., Ignacimuthu S. (2012). In Vitro Antioxidant and Antiproliferative Potential of Medicinal Plants Used in Traditional Indian Medicine to Treat Cancer. Redox Rep..

[32] Turan I., Demir S., Kilinc K., Burnaz N.A., Yaman S.O., Akbulut K., Mentese A., Aliyazicioglu Y., Deger O. (2017). Antiproliferative and Apoptotic Effect of *Morus nigra* Extract on Human Prostate Cancer Cells. Saudi Pharm. J..

[33] Wu X., Gong S., Roy-Burman P., Lee P., Culig Z. (2013). Current Mouse and Cell Models in Prostate Cancer Research. Endocr. Relat. Cancer.

[34] Aghaei M., Karami-Tehrani F., Panjehpour M., Salami S., Fallahian F. (2012). Adenosine Induces Cell-Cycle Arrest and Apoptosis in Androgen-Dependent and-Independent Prostate Cancer Cell Lines, LNcap-FGC-10, DU-145, and PC3. Prostate.

[35] Roomi M.W., Bhanap B., Niedzwiecki A., Rath M. (2021). A Nutrient Mixture Reduced Tumor Growth of SK-UT-1 Human Leiomyosarcoma Cells in Vivo and in Vitro by Inhibiting Mmps and Inducing Apoptosis. Exp. Oncol..

[36] Guggenheim D.E., Shah M.A. (2013). Gastric Cancer Epidemiology and Risk Factors. J. Surg. Oncol..

[37] Plaskova A., Mlcek J. (2023). New Insights of the Application of Water or Ethanol-Water Plant Extract Rich in Active Compounds in Food. Front. Nutr..

[38] Abubakar A.R., Haque M. (2020). Preparation of Medicinal Plants: Basic Extraction and Fractionation Procedures for Experimental Purposes. J. Pharm. Bioallied Sci..

[39] Althwanay A., Alharthi M.M., Aljumaan M., Almubarak Y., Alamri A. (2020). Methanol, Paracetamol Toxicities and Acute Blindness. Cureus.

[40] Saleh K.A., Albinhassan T.H., Al-Ghazzawi A.M., Mohaya A., Shati A.A., Ayoub H.J., Abdallah Q.M. (2020). Anticancer Property of Hexane Extract of *Suaeda fruticose* Plant Leaves against Different Cancer Cell Lines. Trop. J. Pharm. Res..

[41] Somaida A., Tariq I., Ambreen G., Abdelsalam A.M., Ayoub A.M., Wojcik M., Dzoyem J.P., Bakowsky U. (2020). Potent Cytotoxicity of Four Cameroonian Plant Extracts on Different Cancer Cell Lines. Pharmaceuticals.

[42] Harborne J.B. (1973). Phytochemical Methods a Guide to Modern Techniques of Plant Analysis Book.

[43] Trease G.E., Evans W.C. (1989). Pharmacognsy.

[44] Sofowora A. (1993). Phytochemical Screening of Medicinal Plants and Traditional Medicine in Africa.

[45] Edeoga H.O., Okwu D.E., Mbaebie B.O. (2005). Phytochemical Constituents of Some Nigerian Medicinal Plants. Afr. J. Biotechnol..

[46] Mashile G.P., Mpupa A., Nomngongo P.N. (2018). In-Syringe Micro Solid-Phase Extraction Method for the Separation and Preconcentration of Parabens in Environmental Water Samples. Molecules.

[47] Madikizela B., McGaw L.J. (2019). In Vitro Cytotoxicity, Antioxidant and Anti-Inflammatory Activities of *Pittosporum viridiflorum* Sims and *Hypoxis colchicifolia* Baker Used Traditionally against Cancer in Eastern Cape, South Africa. S. Afr. J. Bot..

[48] Wintola O.A., Olajuyigbe A.A., Afolayan A.J., Coopoosamy R.M., Olajuyigbe O.O. (2021). Chemical Composition, Antioxidant Activities and Antibacterial Activities of Essential Oil from *Erythrina caffra* Thunb. Growing in South Africa. Heliyon.

[49] Mosmann T. (1983). Rapid Colorimetric Assay for Cellular Growth and Survival: Application to Proliferation and Cytotoxicity Assays. J. Immunol. Methods.

